# Supplementary figures and images for: Elucidating the complex membrane binding of a protein with multiple anchoring domains using extHMMM
Source: PLoS Comput Biol. 2024 Jul 8;20(7):e1011421. doi: 10.1371/journal.pcbi.1011421 (PMC11257402; doi:10.1371/journal.pcbi.1011421)

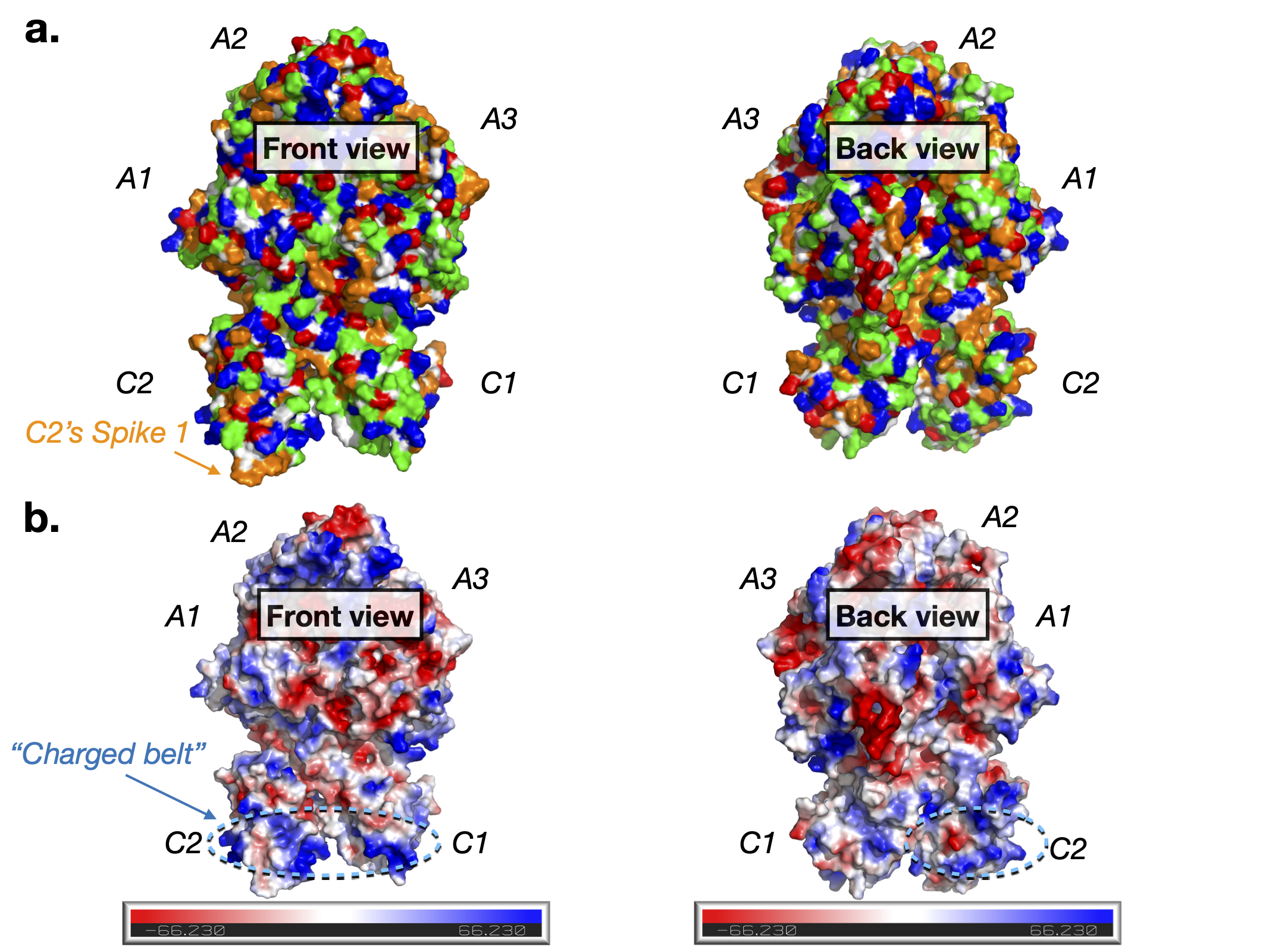

Supplement: S1 Fig — (a) Representation with color-coding based on residue type, showing the exposed protein backbone (in white), positive residues (in blue), negative residues (in red), polar residues (in green), and hydrophobic residues (in orange). The arrow indicates the position of C2’s spike 1 residues W2063 and W2064. (b) Representation showing the electrostatic surface of the FVa molecule, computed using PyMOL’s vacuum electrostatics functionality. The emphasized regions highlight the location of the “charges belt” of positive residues on C1 and C2. (TIFF) [file pcbi.1011421.s001.tiff]

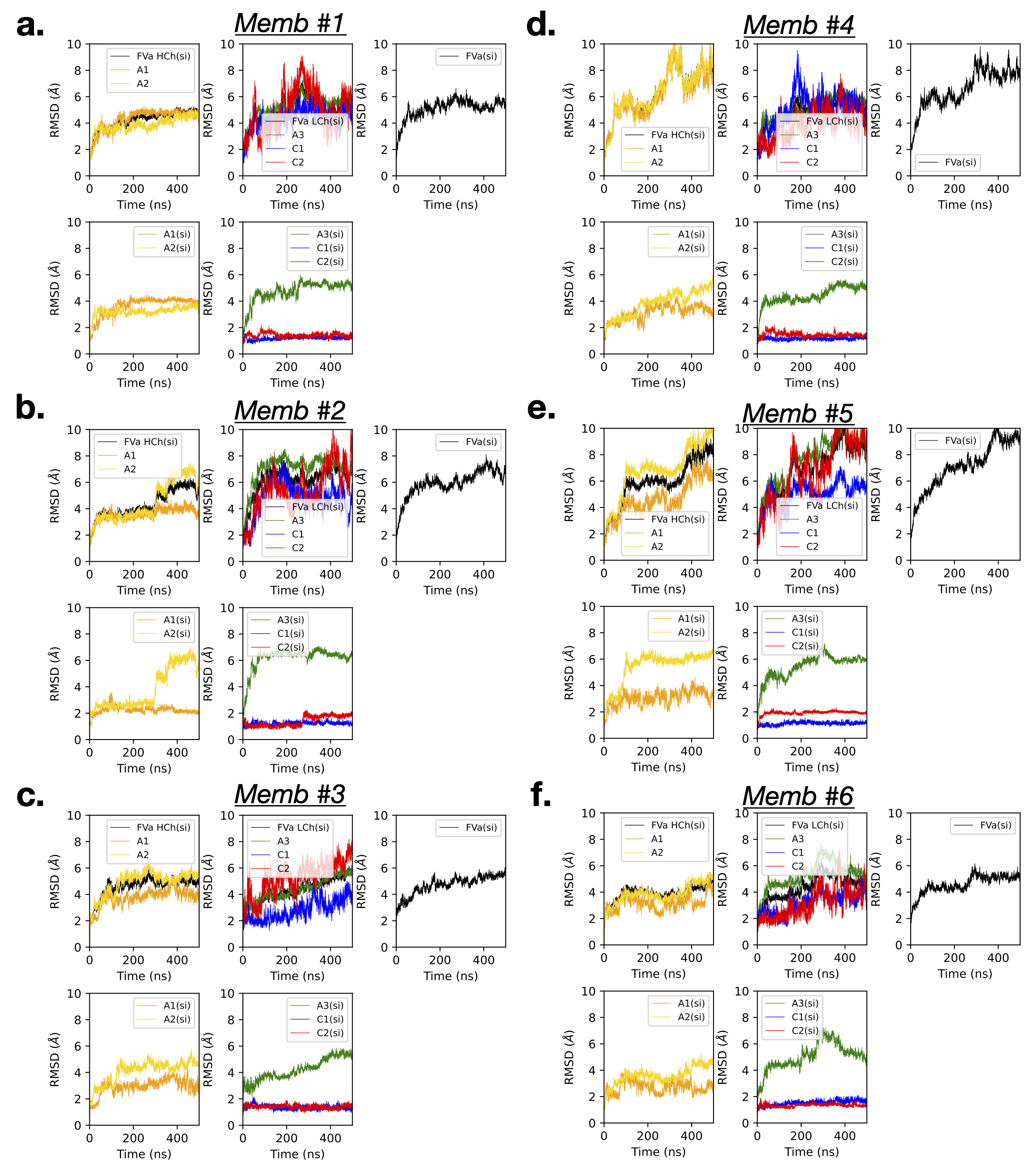

Supplement: S2 Fig — The different panels include RMSD calculation for multiple superimposed selections each corresponding to a specific superimposed alignment applied before calculating the RMSD, enabling comparison between both the different simulations and domains of FVa. The term “si” indicates which selection was superimposed before RMSD calculation. (TIFF) [file pcbi.1011421.s002.tiff]

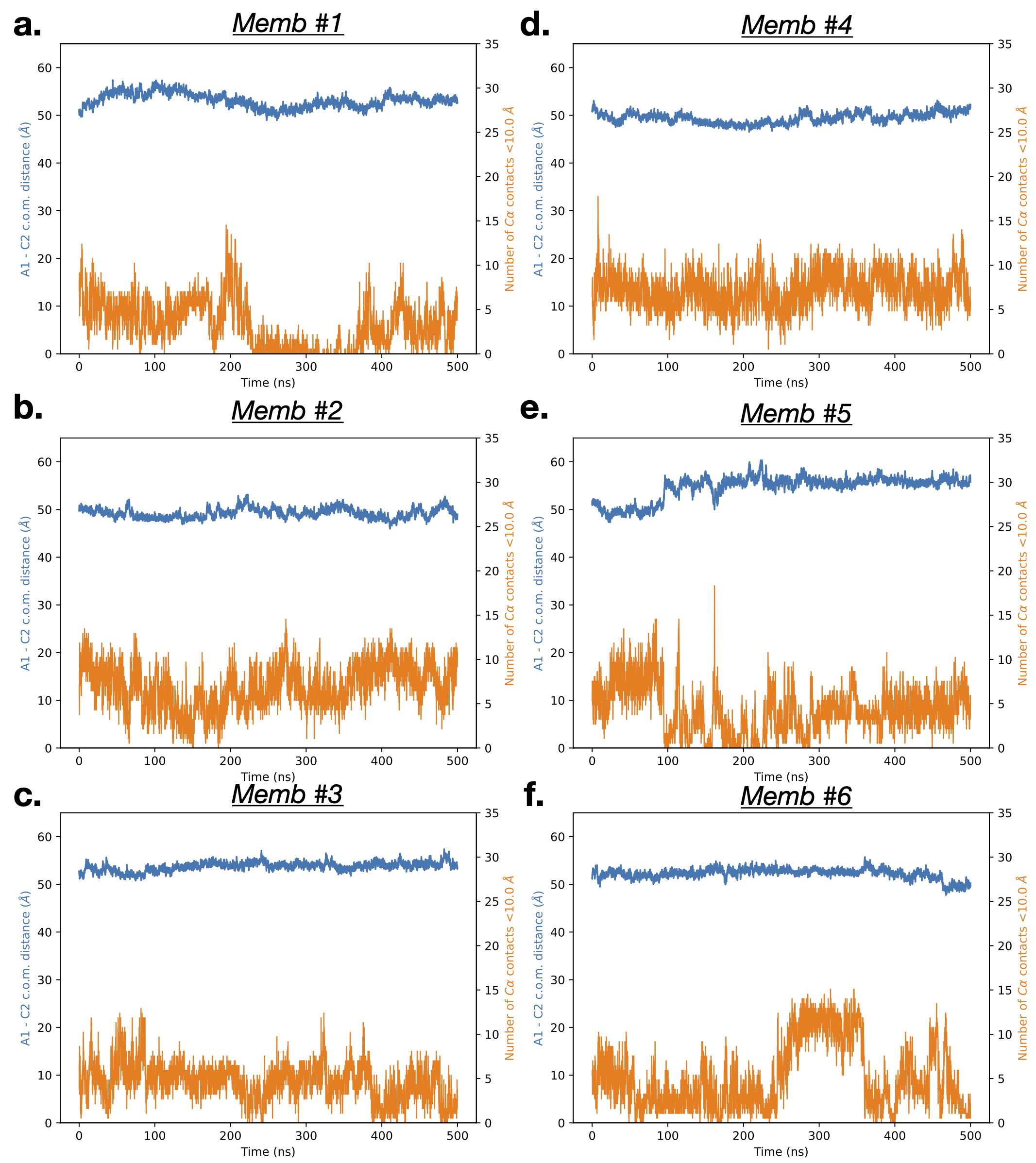

Supplement: S3 Fig — The number of contact points (Cα distance within 10.0 Å) between residues of the A1 and C2 domains as well as the A1 and C2 center of mass (c.o.m.) inter-domain distance in the membrane-containing simulations (Memb #1-#6), both measures of the dynamic interactions between these domains during the simulations. (TIFF) [file pcbi.1011421.s003.tiff]
