# Supplementary material for: Elucidating the complex membrane binding of a protein with multiple anchoring domains using extHMMM
Source: PLoS Comput Biol. 2024 Jul 8;20(7):e1011421. doi: 10.1371/journal.pcbi.1011421 (PMC11257402; doi:10.1371/journal.pcbi.1011421)
Supplement: Attachment — Submitted filename: Review Elucidating the complex membrane binding of a protein with multiple anchoring domains.pdf [file pcbi.1011421.s004.pdf]

Review: Elucidating the complex membrane binding of a protein with multiple anchoring domains

Summary

The article is a molecular dynamics (computational) study of Coagulation factor Va (FVa) binding to lipid membranes. The authors propose that the binding domain reorganises, from it's crystallographic structure, on membrane binding and that this is coupled with a change from 'upright' to 'tilted' configuration. Furthermore they propose that specific amino acids exhibit contact preference to phosphatidylserine lipids compared to phosphatidylcholine lipids. The aforementioned preference seems to be driven by electrostatic preferences.

The authors propose that the results might have therapeutic insight.

The authors propose an amendment/improvement to HMMM to accommodate simulations of large proteins like Fva. This seems to be a key conclusion from the work, and perhaps doesn't have enough discussion of the implications.

It appears that the simulations were performed close to best-practice, and well analysed. I think that there are some interesting observations, that need to be more clearly reconciled with existing publications documenting experimental evidence that agrees or disagrees with this study.

Recommendations

Introduction

It would be helpful to the reader to unambiguously define the FVa being studied without needing to refer to article 29 (Shim et. al). i.e. which Uniprot ID describes the protein.

It would be interesting to see a citation that discusses the therapeutic potential for better understanding Fva binding. Or some more specific recommendation in the discussion. Otherwise this comment should be removed.

In the introduction it wasn't clear to me if anyone already did MD simulations of the Fva membrane system. This could be made explicitly clear either way.

Materials and Methods

What is the thinking behind the initial orientation? Experimental evidence for binding by certain residues? Electrostatics suggesting that is likely to be the preferred orientation?

I didn't see clearly stated how many simulations were performed and for what length.

Since the trajectories are deposited at Zenodo it would make sense to include the analysis scripts there as well.

It would be informative to have a short estimate of used computational time to perform these simulations. i.e. x cpu hours.

Results

The section Mode of Fva membrane binding first presents the results of MD simulations, and then finishes with a discussion of the relation to existing models and experiments. This might be more suited to the Discussion section. In the discussion should also be compared to results from: The factor V C1 domain is involved in membrane binding: identification of functionally important amino acid residues within the C1 domain of factor V using alanine scanning mutagenesis - PubMed (nih.gov)

A schematic figure showing the order of residue binding might be very helpful, perhaps also a table/graph that shows the time variability between the insertions in different trajectories.

It would be helpful to have electrostatic and hydrophobicity plots of the protein structure. This would help to justify the initial configuration, and the likelihood that the described embedding into the membrane is most likely to occur exclusively as described.

It wasn't clear to me what the definition of the 'solution' structures of Fva are. From other work, this work, or from a setup not described in the methods? I think I figured this out in Table 1, but I still think that doesn't tally with the methods.

Discussion

The discussion needs to be clearly linked to existing experimental results, and additionally to perform an additional literature search to check nothing is missed (e.g. the relevant publication referred to in the results section).

It would be relevant to compare the results obtained here to more simple structural bioinformatic methods. Could similar conclusions have been drawn with significantly less computational resources and time? e.g. MBPpred - Home Page (uoa.gr) or Membrane contact probability: An essential and predictive character for the structural and functional studies of membrane proteins | PLOS Computational Biology

Figure 4

I'd consider changing the colours from red/blue to something else for contact frequencies as it can be confusing as this colour scheme is conventionally used for electrostatics.
