# Supplementary material for: Elucidating the complex membrane binding of a protein with multiple anchoring domains using extHMMM
Source: PLoS Comput Biol. 2024 Jul 8;20(7):e1011421. doi: 10.1371/journal.pcbi.1011421 (PMC11257402; doi:10.1371/journal.pcbi.1011421)
Supplement: Attachment — Submitted filename: PLOSCB.rebuttal.020324.docx [file pcbi.1011421.s005.docx]

------------------------------------------------------------------------------------------------------------------------------

**Reviewer 1**

Reviewer #1: Uploaded as an attachment.

**Introduction**

It would be helpful to the reader to unambiguously define the FVa being studied without needing to refer to article 29 (Shim et. al). i.e. which Uniprot ID describes the protein.

Please note that ref. 29 is mentioned because of the procedure FVa was modeled structurally with respect to missing loops and post-translational modifications. We mention in the manuscript that the structure is that of human coagulation factor Va (in Materials & Methods) and in the revision we have added the corresponding UniProt entry name as well.

It would be interesting to see a citation that discusses the therapeutic potential for better understanding Fva binding. Or some more specific recommendation in the discussion. Otherwise this comment should be removed.

Controlling the membrane binding of FVa leads to controlling coagulation. As mentioned in the section, FV is located at the intersection of the intrinsic and extrinsic pathways, making FV more important than some other factors. We added a few references here in which specific strategies are discussed at some length.

In the introduction it wasn’t clear to me if anyone already did MD simulations of the Fva membrane system. This could be made explicitly clear either way.

We modified the text accordingly, including citing Orban et al. Biochemistry 2005; 44,13082 (<https://doi.org/10.1021/bi050891t>). In addition, we discuss some simulation studies from the homologous FVIIIa molecule, e.g., Du et al. Thromb Harmost 2015; 113, 373 and Li et al. J Colloid Interface Sci 2022; 608, 435, and our own previous work.

**Materials and Methods**

What is the thinking behind the initial orientation? Experimental evidence for binding by certain residues? Electrostatics suggesting that is likely to be the preferred orientation? I didn’t see clearly stated how many simulations were performed and for what length.

The performed simulations are listed in Table 1. However, we added the ‘six’ explicitly in the Method and Materials and Results sections for better readability. We agree with the reviewer that the initial orientation is an important aspect, and we have modified the text accordingly to make our justifications clear to the readers. In short, since FVa is known to bind to the membrane through its C1/C2 domains, aligning FVa appropriately with respect to the membrane is beneficial, in order to restrict the search space and minimize irrelevant detours. The choice of a 1-nm separation is also reasonable, as at this distance FVa just barely feels the presence of the membrane – not enough to distort the binding but just enough to guide it toward the membrane. Coarser-grained modeling (e.g., such as Martini) would enable for a more comprehensive and ‘global’ exploration of the various relative orientations of the protein to the membrane; However, we stress that the power of our approach is that we can maintain the atomistic detail of the simulation and thus a more faithful description of the molecular dynamics.

Since the trajectories are deposited at Zenodo it would make sense to include the analysis scripts there as well.

Our analysis scripts are straightforward applications relying on well-established and well-documented codebases cited accordingly. While they are not included directly with the trajectories on Zenodo, we address this point in the manuscript by making them available upon request from the authors. We believe this approach follows accepted standards.

It would be informative to have a short estimate of used computational time to perform these simulations. i.e. x cpu hours.

We achieve performance of ~15ns/day using the following architecture: 24 cores, 2x Intel® Xeon® Silver 4214 CPU @ 2.20GHz with 3x RTX 2080 Ti GPUs for the membrane systems. This detail is added to Materials & Methods.

**Results**

The section Mode of Fva membrane binding first presents the results of MD simulations, and then finishes with a discussion of the relation to existing models and experiments. This might be more suited to the Discussion section. In the discussion should also be compared to results from: The factor V C1 domain is involved in membrane binding: identification of functionally important amino acid residues within the C1 domain of factor V using alanine scanning mutagenesis - PubMed (nih.gov)

The relevant sentences are relocated to Discussion, and some additional remarks are added. However, since only the wild-type FVa construct is studied here, we only discuss FVa mutants insofar that they can help rationalize observed behavior of wild-type FVa.

A schematic figure showing the order of residue binding might be very helpful, perhaps also a table/graph that shows the time variability between the insertions in different trajectories.

It would be helpful to have electrostatic and hydrophobicity plots of the protein structure. This would help to justify the initial configuration, and the likelihood that the described embedding into the membrane is most likely to occur exclusively as described.

We have added a structural plot of the FVa, showing where the locations of charged, polar, and hydrophobic residues on its surface (Fig. S1). We now also discuss the implications of this surface presentation toward the membrane and how it justifies the initial configuration and detailed interactions with the membrane.

It wasn’t clear to me what the definition of the ‘solution’ structures of Fva are. From other work, this work, or from a setup not described in the methods? I think I figured this out in Table 1, but I still think that doesn’t tally with the methods.

This terminology refers to FVa in aqueous solution (i.e., not bound to the membrane). A short description is added in Materials & Methods for added clarification.

**Discussion**

The discussion needs to be clearly linked to existing experimental results, and additionally to perform an additional literature search to check nothing is missed (e.g. the relevant publication referred to in the results section).

It would be relevant to compare the results obtained here to more simple structural bioinformatic methods. Could similar conclusions have been drawn with significantly less computational resources and time? e.g. MBPpred - Home Page (uoa.gr) or Membrane contact probability: An essential and predictive character for the structural and functional studies of membrane proteins | PLOS Computational Biology

We acknowledge the reviewer’s comment regarding potential use of bioinformatics tools, and recognize the limitations of MD, especially concerning the (computational) time required. Elucidating membrane-bound conformations and molecular interactions between protein and lipid will require a technique of adequate precision and accuracy. While bioinformatics approaches are efficient in identifying potential regions of a protein what would favor various environments (aqueous vs membrane interior), which could provide a picture similar to our results presented in Fig. 4, they are not able to elucidate any of the other results we present in the current manuscript.

Figure 4

I’d consider changing the colours from red/blue to something else for contact frequencies as it can be confusing as this colour scheme is conventionally used for electrostatics.

We added labels to the color bar in Fig. 4c, as in Fig. 5b. Also, ‘red’ and ‘blue’ in the legend are now shown in bold for readability. Similar color-term highlights are applied to all other figure legends.

------------------------------------------------------------------------------------------------------------------------------

**Reviewer 2**

Reviewer #2: Madsen and Ohkubo's research investigates the interaction between the Coagulation factor Va (FVa) protein and a membrane composed of PC and PS (in a 4:1 ratio) through molecular dynamics simulations. They employed a modified version of the highly mobile membrane-mimetic (HMMM) model to achieve this. In this revised model, they extended the length of the lipid chains from 5 to 8 carbons (dioctanoyl). This extension was crucial to creating a sufficiently long hydrophobic core that could accurately replicate the interactions between FVa and lipids, especially when FVa is deeply embedded within the membrane. This innovative approach is clever and significant.

Their study addresses five key aspects:

i) Investigating the binding motif mechanism of FVa's multiple anchoring domains.
ii) Analyzing the tilting behavior of the protein when it binds to the membrane.
iii) Comparing the structural disparities between the crystallographic structure and the model in both the membrane and aqueous environments.
iv) Characterizing the interactions between lipids and the protein.

This research represents a noteworthy contribution to understanding the complex interactions between FVa and lipid membranes, shedding light on important aspects of this biological process.

We thank the reviewer for the encouraging comments.

However, I have several concerns regarding the construction of the systems, which may not be conducive to a comprehensive study of the binding mechanism (point (i) ). In particular, how the systems were built seems inadequate for addressing the mode of FVa membrane binding. Systems were constructed with the C2 domain facing the membrane at a distance of 1nm, while the cutoff values for coulombic and van der Waals interactions are set at 1.2. Consequently, these components are not at a non-interacting distance as one would expect. Therefore, the author's claim that "Initial contact and insertion of the C2 and C1 domain spikes progress sequentially" is not surprising, given the bias in their system. Biased initial conditions influence the initial systems.

We agree with the reviewer that this an important aspect, and we have modified the text accordingly to make our justifications clear to the readers. In short, since FVa is known to bind to the membrane through its C1/C2 domains, aligning FVa appropriately with respect to the membrane is beneficial, in order to restrict the search space and minimize irrelevant detours. The choice of a 1-nm separation is also reasonable, as at this distance FVa just barely feels the presence of the membrane – not enough to distort the binding but just enough to guide it toward the membrane. Coarser-grained modeling (e.g., such as Martini) would enable for a more comprehensive and ‘global’ exploration of the various relative orientations of the protein to the membrane; However, we stress that the power of our approach is that we can maintain the atomistic detail of the simulation and thus a more faithful description of the molecular dynamics.

The system is large, but the author could have improved the study's validity by commencing with multiple random orientations where the C2 and C1 domains do not face the membrane. This would have allowed for a more thorough exploration of the binding motif. If the author intends to delve deeper into which of the two domains initiates the binding process, the authors should consider employing umbrella sampling to generate a free energy profile. This additional approach would provide a more comprehensive understanding of the energetic landscape governing the binding of these domains to the membrane. It's important to note that the chosen approach is valid only when the binding motif is already known.

The reviewers’ comment is valid. While we acknowledge the potential benefits of starting from multiple initial orientations of FVa relative to the membrane, we feel that our approach is logical and justifiable in accordance to our reply in the above. We had made some attempts toward pursuing a free-energy profile, but we found it difficult to obtain accurate and meaningful estimations. For a large protein such as FVa, in particular, an appropriate measure (C.V.) to include all important orientations and distances with respect to the membrane is complex and sampling expensive. In addition, the membrane itself fluctuates and the lateral composition is heterogeneous and dynamic, which complicates matters further. In short, we do not know of any robust way to approach this pursuit.

Given the simulations' limitations, it is impossible to derive any insights about the binding mechanism, specifically whether it involves C2 or C1 first. Consequently, any statements regarding the binding mechanism (C2 vs C1) should be omitted from the discussion to maintain scientific accuracy.

Please note that we present some necessary conditions for a certain membrane-binding form of FVa, rather than claiming sufficiency, which would be challenging both for experimental and computational methods to achieve. However, we appreciate the reviewer’s comment.

**Major comments:**

1) Furthermore, the methodology employed for constructing the initial structures needs more clarity. Did the author generate random initial velocities, or were six separate membrane constructions carried out? I could only infer this information by downloading and inspecting the trajectory as a reader. To enhance transparency and reproducibility, such crucial details should be explicitly outlined in the "Materials and Methods" section of the study. Additionally, it would be advantageous to include a concise summary of this information in the Results section, not solely confined to the Methods section.

We agree with the reviewer, and more information were added to Methods, and a short summary of the system preparation was included at the beginning of Results.

2) The results presented in Figure S1 and S2 require more comprehensive explanations. The authors mention what has been calculated, but what specific findings have been derived from the data needs to be clarified. The figure displays RMSD values without effectively highlighting the key results or insights obtained. Additional context and interpretation of the figure's data would enhance its clarity and usefulness. Furthermore, I couldn't discern any notable differences in the A1-C2 distance. Maybe, it would have been beneficial to generate a two-dimensional histogram encompassing all trajectories to address this issue. This histogram could have depicted the relationship between the center of mass (COM) distance and the number of contacts, shedding light on whether a correlation exists between a high number of contacts and a shorter distance (and vice versa). This is just a suggestion; I want to mention you can display the result differently.

More extensive explanation is added to describe the results of shown in Figs. S1 and S2 (now S2 and S3). The RMSD plots are shown to indicate that the intradomain structural deviations are negligible, except for the A3. A global alignment (top right panel) covers up much of what is going on. For instance, alignment of the heavy chain readily allows one to track whether C1 or C2 is moving relative to the complex when they rearrange relative to each other.

We agree with the reviewer's observation that there is no correlation between the distance of the A1-C2 center of mass and the count of inter-domain contacts. This was verified through examining the 2D scatterplot as suggested. Our conclusion is that although C2 cannot detach from the complex, the relative movements do indeed change the nature of the interaction with the adjacent domains.

3) The absence of substantial differences between PS and PC contacts in Figure 4a is noteworthy. The authors attribute this observation to the stoichiometric ratio of the bilayer, PC: PS=4:1. While this hypothesis appears plausible, it can be supported by calculating the contact frequency while normalizing it against the total number of lipids. Furthermore, there is a need for clarity in explaining how the frequency of contact is calculated and subsequently normalized. This crucial procedure needs more specification in the figure caption and/or the Material and Methods section. Additionally, it is crucial to address whether any portions of the trajectory have been excluded from the analysis, as this information is presently only indicated in Figure 6. Furthermore, error bars are missing.

It seems to be a matter of choice of the target against what the data is normalization: actual contact frequencies or those normalized against the lipids of the same kind.

4) In addition to the contact frequency analysis, exploring the interaction energies, encompassing both van der Waals (vdW) and Coulombic interactions could be highly beneficial between PC and PS. Such an investigation will likely reveal substantial disparities in these interactions, offering further insights into the nature of the interactions.

We have added a structural plot of the FVa, showing where the locations of charged, polar, and hydrophobic residues on its surface (Fig. S1). We now also discuss the implications of this surface presentation toward the membrane and how it justifies the initial configuration and detailed interactions with the membrane.

5) Figure 6 exhibits high distribution of data, making it challenging to draw conclusive findings. The issue may stem from including excessive frames and incorporating all trajectories. To address this concern effectively, the author should consider classifying the orientations, possibly based on tilting, and subsequently analyze and average frames with similar orientations. This approach will likely enhance the results' reliability and provide a more robust basis for conclusions. Same approach can be used in aother analysis too.

The decision to average the analyses over trajectories and states was made deliberately to compute ensemble-averaged properties that could potentially be compared with experimental observations. We acknowledge the comment on the distribution of data in Figure 6 and appreciate the constructive input. Still, we believe that deconvoluting the analysis might introduce unnecessary complexity and adding confusion.

6) Regarding the use of two different rAPLs, the study does not explain, nor does it discuss the unique characteristics, benefits, drawbacks, or potential effects on the results. It remains unclear whether specific features, such as tilting, contacts, and binding motifs, are influenced by rAPL. Averaging all trajectories together might not be the right choice since systems might behave differently.

We agree with the reviewer. In short, one of the reasons is that we are interested in the critical APL (as well as acyl tail length) at which the membrane lipid dynamics are significantly increased from those in nature, while still being faithful with regards to lipid headgroup structure and lipid-protein interaction. Clearly, there is a trade-off between the lipid dynamics and accuracy: the larger APLs and shorter tail length leads augmented dynamics but lesser accuracy of lipid properties. Furthermore, one might expect that protein insertion depth could vary with rAPL while we did not find this for our current setup. We think that there may still be room to improve the extHMMM with regard to these specific parameters, in particular for certain applications. Explanations and discussion of the reasoning for two rAPLs were added to the manuscript text.

7) I noticed an unusual structure in the Discussion section. The authors are more focused on justifying the importance of their study by comparing it to other proteins and mechanisms rather than delving into interpreting their results. It gives the impression of a review-style format with multiple paragraphs, each starting with an introduction. Instead, a more concise approach is advisable. The primary focus should be on discussing their work, with a brief concluding section addressing the potential relevance to other proteins. Similarly, the Comments section on modifying the HMMM model could be integrated into the main discussion without requiring a separate header. Openly discussing these points within the context of the broader discussion would create a more cohesive and streamlined narrative.

We have chosen to divide Results and Discussion sections in order to separate our speculations interpretations lacking direct support from the current results presented in the manuscript or those arising from multiple results. This division allows us to be precise when describing our results. However, we have attempted to the best of our ability to revise the entire manuscript to make it clear and streamlined.

**Minor comments:**

- In the Material and Methods section, there is mention of an "NPnAT ensemble." Is it a typo?

A short explanation is added in parenthesis after the term. NPnAT, sometimes also denoted as NPzAT refers to pressure control in the z direction – i.e. the membrane normal.

- In Figure 4d, it would be beneficial to explicitly specify which section of the membrane is represented by the dotted lines for clarity.

Labels are added to Fig. 4c.

- The majority of the references conclude with the notations "From NLM PubMed-not-MEDLINE" and "From NLM Medline." Consider removing it.

All removed.

------------------------------------------------------------------------------------------------------------------------------

**Reviewer 3**

Reviewer #3: In this paper the authors have discussed the complexities of protein-lipid interactions in the context of FVa. They use a new molecular dynamics method termed “extHMMM” to identify key mechanisms of FVa binding to the membrane such as molecular tilt, domain organization, and contact preferences. The reviewer recommends the article be published after minor revision. The reviewer has the following comments:

1. Abstract

There are two typos in the abstract.

Corrected.

2. Introduction

The “extHMMM” model is very important for the basis of this study, the reviewer suggests adding more background on the model in the introduction paragraph.

Edited as suggested. We now include the term ‘extHMMM’ in the introduction and title of our manuscript.

3. Results: Mode of FVa membrane binding

The reviewer suggests further discussion of the following topics: What are the further differences between the binding patterns of FVa to membrane surfaces and those observed in lipid-decorated nanotube models, and what implications does this have for understanding how FVa interacts with regular cell membranes functionally?

This is an important issue with some associated controversy. We have discussed this a bit further modified the relevant paragraphs. Basically, our data presented here in consistent with our earlier studies and the “deeply inserted” binding modes described in assays involving lipid-decorated carbon nanotubes are not seen. A step toward resolving this would be to compute the free energy profile along a path connecting the two states, but this is beyond the scope of our current manuscript.

4. Results: Domain organization of FVa significantly deviates from the crystallographic reference structure both when membrane-bound state and in solution state, with deviation being more pronounced in the solution state

The reviewer suggests further discussion of the following topics: In the context of FVa's membrane binding how do the observed variations in the φ and θ angles of FVa in solution state and membrane-bound state relate to its potential functional significance?

Given the flexible nature of the relative C2 orientation of the rest of the FVa molecule, observed movements are consistent with previous reports of simulations of the FVa/FVIIIa light chain using different force fields (e.g., Du et al. Thromb Harmost 2015; 113, 373 and Li et al. J Colloid Interface Sci 2022; 608, 435; both cited and discussed) and therefore probably not an simulation artifact. Further, there is experimental evidence of large structural deviation between C1 and C2 by means of AFM (Chaves et al, cited and discussed). We are not aware of any explicit evidence linking the domain organization of the membrane-bound state with functional significance beyond what we have noted in the manuscript already.

5. Results: Certain amino acid residues exhibit contact preference toward interacting with PS lipids

The reviewer suggests further discussion of the following topics: How does discoidin domain absence affect FVa's membrane-bound conformation and interaction with lipid bilayers? How might this lack of deep insertion influence the functional role of FVa in membrane binding? Is there a time-scale difference between first contact with PS lipids over PC lipids before complete insertion?

Our current study does not dig into the impact of how absence of discoidin domains might affect the membrane-bound conformation and FVa interaction with bilayer membrane. We have discussed the functional aspects of the FVa membrane-bound state thoroughly in the manuscript. In particular, we note that deep insertion has been observed exclusively in association with lipid-decorated carbon nanotubes, and we would guess that their functional significance is limited.

6. Results: The spikes of the discoidin domains insert into the hydrophobic region of the lipid membrane.

The reviewer suggests further discussion of the following topics: What is the effect of the depth of insertion of spike residues (e.g., W2063, W2064, L2116, R2080) into the hydrophobic core of the bilayer on anchoring and stability of discoidin domains? Are there functional implications associated with varying insertion depths for different spikes? Do these spikes show changes in structures upon insertion?

The overall structure of the discoidin domain remains relatively stable, as indicated by the RMSD calcuations shown in Fig. S1 (lower middle plots of each panel show that RMSD for C1/C2 are <2 Å in all cases). However, it is worth noting that, despite some discussions about subtle features, such as the “open” and “closed” spike conformations (which we indeed consider as relevant to the mechanism of FVa’s membrane binding), these effects are better elucidated in studies of individual discoidin domains. In such studies, transitions and equilibrium distributions can be thoroughly explored due to the smaller system size, as exemplified by Wu et al. PRE 2009; 79, Ohkubo & Tajkhorsid Biophys J 2013; 104, 432a, and Madsen et al. Biochemistry 2015; 54, 6123. Our present investigation does not significantly contribute to the existing knowledge on this matter.

7. Discussion

Could you elaborate on the implications of the membrane potentially acting as an allosteric effector of FVa? Are there experimental studies that support the concept of membrane allostery in coagulation proteins?

Our interpretation is based on the findings presented in our study, where such an effect would be consistent with the results. We emphasize this as speculative in nature, and we present it as such. Further research would be required to validate and expand upon these observations.

------------------------------------------------------------------------------------------------------------------------------

We also made the following modifications aside from the above. These are noted at the end of the individual figure legends with the ‘(Author note)’ label.

Fig. 1. Inset

Labels for Spikes in C1 are corrected.

Figs. 4 & 5

The rotation marks for FVa are modified to be more intuitive.

Fig. S2 (originally Fig. S1)

Minor modifications on the y-axis and plot labels for reader-friendliness.
