# Supplementary material for: Elucidating the complex membrane binding of a protein with multiple anchoring domains using extHMMM
Source: PLoS Comput Biol. 2024 Jul 8;20(7):e1011421. doi: 10.1371/journal.pcbi.1011421 (PMC11257402; doi:10.1371/journal.pcbi.1011421)
Supplement: Attachment — Submitted filename: rebuttal.R2.docx [file pcbi.1011421.s006.docx]

*Reviewer #1: All but one of my concerns is addressed. I would strongly recommend to open source the analysis scripts. While they may be 'straightforward applications', it is impossible to quickly reproduce the results without them, and making the code available on request adds an unnecessary barrier to accessing the code, and increases the chance that at some point in the future it is lost.*

Our analysis scripts have been open-sourced. The files can now be found at GitHub (<https://github.com/jjmadsen/pub-fva-hmmm.git>).

*Reviewer #2: The authors have addressed my questions and comments to my satisfaction overall. However, a significant issue I raised still needs to be addressed.

The authors introduced the following new text in the Materials and Methods section, which I found incorrect.

"For the membrane system, the protein model is initialized above the membrane with its principal axis aligned with the membrane normal and discoidin
domains facing the membrane at an initial distance of ~1 nm between the membrane surface and the closest α-Carbon so that only a few atoms of FVa have near-zero
electrostatic energies with membrane lipids and the vast majority of FVa atoms have no interaction with the membrane. This initial positioning minimizes initial configuration dependency of the membrane binding processes (if observed), while effectively trim irrelevant detours, given that experiments indicate C1 and C2 are membrane-binding domains".

Although the authors' initial setup successfully eliminates unnecessary deviations, it is inaccurate to claim that "This initial positioning minimizes initial configuration dependency of the membrane binding processes ". In reality, the methodology introduced by the authors imposes a significant bias on the membrane binding processes.*

This sentence was rephrased accordingly to “This initial positioning guides C1 and C2 to bind the membrane, consistent with experiment. Thus, some flexibility is maintained while reducing initial configuration-dependency of the membrane binding processes within the experimentally established framework.” *I agree with the authors' reasoning for their chosen methodology, recognizing it as logical and well-founded. However, their analysis cannot be used to clarify how the protein's binding motif transitions from an aqueous environment to the membrane. Consequently, I suggest revisions to the section titled "Mode of FVa Membrane Binding."

I suggest relocating this section to the Materials and Methods or the Supplementary Information to detail the construction of the membrane-bound state utilized in subsequent analyses. The rationale for this suggestion stems from the observation that the claims within this section lack statistical support and merely describe visual observations from the simulations. Providing a more precise explanation of the methodology in a more appropriate section would enhance the clarity of the subsequent research findings.*

We feel that this information is better kept in the main text of the manuscript (as opposed to the SI). While these observations stem from our specific simulations and could be considered anecdotal, we’ve integrated them within the boarder context of relevant literature. We anticipate that readers interested in FVa would find these observations valuable.

As for the limitation mentioned by the reviewer regarding our analysis’ inability to fully elucidate the binding mode, we feel that the first part of the revised section in question addresses this concern. We explicitly acknowledge variation among trajectories and stress that our setup is biased toward the experimentally established framework.

We thank both reviewers for their comments and suggestions in reviewing our manuscript.
